# Can a diet rich in Brassicaceae help control *Helicobacter pylori* infection? A systematic review

**DOI:** 10.3389/fmed.2024.1454902

**Published:** 2024-12-17

**Authors:** Sara Properzi, Fabrizio Stracci, Margherita Rosi, Chiara Lupi, Anna Villarini, Alessio Gili

**Affiliations:** ^1^Department of Medicine and Surgery, University of Perugia, Perugia, Umbria, Italy; ^2^Department of Life Sciences, Health and Health Professions, Link Campus University, Rome, Italy

**Keywords:** *Helicobacter pylori* infections/diagnosis, *Helicobacter pylori*, Brassicaceae plants, glucosinolates, thiocyanate

## Abstract

**Introduction:**

*Helicobacter pylori* (*Hp*) infection is highly prevalent globally and poses a significant public health challenge due to its link with chronic gastritis, peptic ulcers, and gastric malignancies. *Hp*’s persistence within the gastric environment, particularly in case of infection with virulent strains, triggers chronic inflammatory responses and mucosal damage. Antibiotic therapy is the primary approach for *Hp* eradication, but antibiotic resistance and adverse effects hinder treatment efficacy. Emerging evidence suggests that Brassicaceae-derived metabolites could serve as adjunctive therapy for *Hp* infection, offering potential antimicrobial and anti-inflammatory benefits.

**Methods:**

A systematic literature review was conducted following PRISMA guidelines to assess the impact of Brassicaceae-rich diets on *Hp* infection control. Searches were performed in MEDLINE PubMed, Web of Science, and the Cochrane Library until 18 October 2023, without language or date restrictions. Eligible studies meeting PICOS criteria were included, encompassing populations infected with *Hp* or *Hp*-infected human cell cultures, interventions involving Brassicaceae consumption or its bioactive molecules, and outcomes related to *Hp* infection control, antibiotic therapy interactions, reduction of antibiotic side effects, and inflammation mitigation. Animal studies, cell line experiments, reviews unrelated to the research objectives, and studies on *Hp*-related gastric cancer were excluded.

**Results:**

Available evidence indicates that Brassicaceae consumption exhibits the potential to reduce *Hp* colonization but achieving complete eradication of the pathogen remains challenging. Conflicting results regarding the efficacy of broccoli in *Hp* treatment emerge, with certain investigations suggesting limited effectiveness. Other studies point to a potential for heightened eradication rates when combined with standard triple therapy. Furthermore, promising outcomes are observed with broccoli extract supplements, indicating their role in mitigating *Hp*-induced gastric mucosal damage. In fact, it is noteworthy that sulforaphane and its derivatives manifest notable reductions in pro-inflammatory markers, indicative of their anti-inflammatory properties. Adverse events associated with antibiotic therapy seem unaffected by sulforaphane derivatives or probiotics. However, individual responses to these treatments vary, underscoring the unpredictability of their efficacy in ameliorating antibiotic therapy-related side effects.

**Conclusion:**

Our systematic review highlights the potential of Brassicaceae-rich diets as adjunctive therapy for *Hp* infection, offering synergistic interactions with antibiotics and possibly mitigating antibiotic side effects and inflammation. Further research, particularly well-designed randomized trials, is warranted to elucidate the therapeutic efficacy and optimal utilization of Brassicaceae-derived metabolites in managing human *Hp*-related diseases.

## 1 Introduction

*Helicobacter pylori* (*Hp*) is a Gram-negative microaerophilic bacterium (1), that colonizes one-half of the world’s population (2). *Hp* is the main risk factor for gastric cancer (3). Disseminating through oro-oral and/or oro-fecal routes (4), generally within families during early childhood (2), before the age of 10 years (5). However, high prevalence and early age of infection do not always translate in high incidence of gastric cancer (6). Risk and protective factors may modulate gastric cancer risk in *Hp* infected patients and consumption of Brassicaceae has been proposed as a possible protective factor (7).

Over the past century, there has been a remarkable but not uniform global reduction in the prevalence of *Hp* infection. Recent studies indicate a significant decrease in the global prevalence of *Hp* infection from 58% in the 1980–1990 period to 43% in 2011–2022 (5). However, this overall decline masks substantial regional and demographic variability. The most notable reductions have been observed in high-income countries, reflecting sanitation, healthcare access, spread of *Hp* eradication treatment, and other public health initiatives. In contrast, the prevalence remains high in many developing countries, particularly in parts of Africa, South America, and Asia, where the prevalence of infection often exceeds 70%. These areas experience slower declines due to persistent socioeconomic challenges, limited access to clean water, and crowded living conditions, which facilitate the transmission of *Hp* (8).

Moreover, in high-income countries, younger populations exhibit significantly lower prevalence rates compared to older cohorts, reflecting the impact of improved living conditions and healthcare advancements over recent decades (8). Despite the fact that approximately 80% of colonized individuals display no symptoms, persistent *Hp* infection almost always leads to the development of chronic gastritis (9). The severity of the chronic inflammatory process associated with *Hp* infection is influenced by various factors, including the colonizing strain’s virulence, host genetics, immune response, and dietary habits (10). The disease spectrum associated with *Hp* chronic infection encompasses duodenal ulceration, often associated with antral gastritis, gastric ulceration in cases of corpus gastritis, gastric malignancy (10), and MALT (Mucosa Associated Lymphoid Tissue) lymphoma (11–13). *Hp*’s survival and proliferation within the acidic gastric milieu is facilitated by its secretion of urease, which enzymatically hydrolyzes host urea into ammonia and nitric oxide (14) and facilitates flagellar motility through the mucus layer by changing the viscoelasticity properties of gastric mucins (15). *Hp*’s adhesion and colonization of the gastric mucosa are mediated by adhesins (BabA, blood group antigen-binding adhesion (16), SabA, sialic acid-binding adherence (16), and LabA, lacdiNAc-binding adhesin (17)) and virulence factors like VacA (Vacuolating-toxin A, apoptotic cell death inductor or necrosis inductor through mitochondrial membrane perturbation. VacA suppresses T and B cell proliferation, as well as various immune cell populations, thereby downregulating immune responses to *Hp* infection and promoting host tolerance to the organism (18)) and CagA (Cytotoxin-associated gene A involved in adhesion mechanisms and pro-inflammatory cytokine release (13)), predominantly expressed by more pathogenic *Hp* strains (13). Host receptors (TLR and NOD-like receptors) bind to pathogen components, eliciting pro-inflammatory gene activation (13). This immune response activates the NF-κB (Nuclear factor kappa-light-chain-enhancer of activated B cells) pro-inflammatory signaling pathway (19) and the NRF2 (Nuclear factor erythroid 2-related factor 2) anti-inflammatory signaling pathway (20), leading to cytokine release including TNF-*α*, IL-1β, IL-8, and IFN-*γ* (21).

Following *Hp* colonization, the gastric mucosa undergoes cellular infiltration, primarily consisting of neutrophils and mononuclear cells. These infiltrating cells actively engage in phagocytosis of the pathogen and, by expressing inducible nitric oxide synthase (iNOS), secrete nitric oxide and reactive oxygen species (ROS) (22). In fact, in *Hp*-infected gastric mucosa, neutrophils are believed to be the main source of ROS (23). After the recognition of pathogenic bacteria, neutrophils immediately phagocytize and kill them through ROS (24). However, in the *Hp*-infected gastric mucosa, immune cells cannot eradicate the bacteria. Therefore, a persistent inflammatory status of gastric mucosa is established where the phagocytes produce inflammatory cytokines and ROS in response to bacteria. This excessive production of ROS is believed to be a major cause of gastric mucosal damage (24), and contributes, together with direct mucosal damage from virulent *Hp* strains, to the multi-step pathogenesis of chronic lesions of the stomach (22, 25). Furthermore, *Hp* infection elevates blood levels of Pepsinogen I (PGI), Pepsinogen II (PGII), and Gastrin-17 (G-17), serving as biological markers to assess gastric mucosal inflammation (26) and functionality (27).

An effective treatment regimen for pathogen eradication and control is critical to mitigate the clinical manifestations of *Hp* infection (28). International guidelines advocate assessing local clarithromycin resistance prevalence to select first-line therapy selection. In regions where the prevalence of clarithromycin-resistant *Hp* strains is high (>15%) or unknown, a 14-day quadruple therapy incorporating a proton pump inhibitor (PPI), amoxicillin or bismuth, clarithromycin, and metronidazole is recommended for eradication (29). PPIs are believed to synergize with antimicrobials by attenuating gastric acidity (30), inhibiting *Hp* urease activity (31), and exhibiting intrinsic antibacterial properties (30). It is also known that PPIs can significantly impact the gut microbiota reducing microbial diversity and increasing the risk of overgrowth of harmful bacteria (32).

Furthermore, various natural products and food components exhibit anti-*Hp* activity. Key agents include polyphenols, flavonoids, essential oils, and compounds from medicinal plants. These substances work through mechanisms such as inhibiting *Hp* growth, reducing adhesion to gastric cells, and modulating inflammation (33).

A recent narrative review on *Hericium erinaceus*, commonly known as the Chinese mushroom, underscores the potential of specific mushroom fractions, particularly those derived from ethyl acetate or HEP25/75, which exhibit promising anti-*Hp* activities. These fractions demonstrate noteworthy minimum inhibitory concentration (MIC) values against the bacterium (34).

Previous investigations (35–37) underscore the potential of compounds in Brassicaceae vegetables (e.g., Cabbage, Broccoli, Cauliflower, Brussels sprouts, Kale, Collard greens, Turnip, Radish, Mustard greens, Horseradish, Rutabaga (Swede), Watercress, Arugula (Rocket), Bok choy (Pak choi), Chinese cabbage, Rapeseed (Canola), Wasabi (38)) to mitigate oxidative stress *in vitro* and exert anti-inflammatory effects on *Hp*-infected gastric mucosa in animal models and humans. Cruciferous vegetables, particularly broccoli sprouts, contain glucosinolates, such as glucoraphanin, and quercetin, that are hydrolyzed by myrosinase within the vegetables themselves and by intestinal flora into active molecules (39), isothiocyanates such as sulforaphane, a potent inducer of phase 2 detoxification enzymes (40), implicated in pro-inflammatory cytokine downregulation. Sulforaphane further mitigates inflammation by reducing NF-κB expression and activating the NRF2 pathway (35), exhibiting bacteriostatic and bactericidal effects against *Hp in vitro* and efficacy against extensively antibiotic-resistant *Hp* clinical isolates (41). Sulforaphane has also been studied for its ability to reduce the risk of colorectal cancer (42) and for enhancing the sensitivity of cancer cells to chemotherapy, potentially overcoming chemoresistance (43).

Quercetin, a flavonoid abundant in Brassicaceae (44), exhibits anti-inflammatory and antioxidant properties in rodents via NRF2 upregulation and NF-κB downregulation (45). However, confirmation of quercetin’s anti-inflammatory and *Hp* infection control efficacy, as demonstrated in animal models (46, 47), in human subjects remains lacking. In fact, despite numerous investigations elucidating the impacts of Brassicaceae derivatives on animals or *in vitro* regarding *Hp* infection, scant evidence substantiates their efficacy in human cases of *Hp* infection. This systematic review explores the potential of a diet rich in Brassicaceae vegetables in influencing the clinical outcomes of *Hp* infection in humans. Particularly it investigates possible synergistic interactions between Brassicaceae-derived metabolites and antibiotic therapy for eradication, and the role of such metabolites in alleviating the adverse effects of antibiotics and mitigating *Hp*-induced inflammation.

## 2 Materials and methods

### 2.1 Research strategy

We conducted a systematic literature review, adhering to the PRISMA statement (48), to investigate the potential impact of a diet rich in Brassicaceae on the control of *Hp* infection. The protocol was registered and is available on PROSPERO (CRD42024517622)^1^. Three electronic databases (MEDLINE PubMed, Web of Science, and Cochrane Library) were searched without language or date restrictions, from inception to 18/10/2023 for relevant original articles.

We started the search on MEDLINE and then identified an appropriate syntax for the other databases. Our search strategy included terms related to *Hp* infection, Brassicaceae vegetables, and their bioactive molecules.

The entire search strategy is reported in Table 1.

**Table 1:** Search strategy.

Search engine	Search strategy
MEDLINE/PubMed	From 1000/01/01 to 18/10/2023: (*Helicobacter pylori* OR *helicobacter pylori* infection) AND (quercetin OR sulforaphane OR glucosinolates OR isothiocyanate) AND (Brassicaceae OR Brassica OR cruciferous vegetables OR broccoli OR cabbage OR cauliflower OR Brussels sprouts OR mustard plants OR mustard oil OR sauerkraut OR coleslaw OR collards OR bok choy OR turnip greens OR vegetables OR rocket OR wasabi)
Web of science	From 1000/01/01 to 18/10/2023: Query: TS = ((*helicobacter pylori* OR *helicobacter pylori* infection) AND (quercetin OR sulforaphane OR glucosinolates OR isothiocyanate) AND (Brassicaceae OR Brassica OR cruciferous vegetables OR broccoli OR cabbage OR cauliflower OR Brussels sprouts OR mustard plants OR mustard oil OR sauerkraut OR coleslaw OR collards OR bok choy OR turnip greens OR vegetables OR rocket OR wasabi)).
Cochrane library	From 1000/01/01 to 18/10/2023: (*helicobacter pylori* OR *helicobacter pylori* infection) AND (quercetin OR sulforaphane OR glucosinolates OR isothiocyanate) AND (Brassicaceae OR Brassica OR cruciferous vegetables OR broccoli OR cabbage OR cauliflower OR Brussels sprouts OR mustard plants OR mustard oil OR sauerkraut OR coleslaw OR collards OR bok choy OR turnip greens OR vegetables OR rocket OR wasabi).

### 2.2 Eligibility criteria

Included studies met the following eligibility criteria described as PICOS (48): (1) P (Population): individuals infected with *Hp* regardless of age, gender, health status, and any other variable influenced by the infection and/or human cells *in vitro* infected with *Hp*; (2) I (Intervention): intake of brassicaceae vegetables and their active bioactive molecules; (3) C (Control): standard or non-necessary; (4) O (Outcome): interaction of treatments with eradication antibiotic therapy and/or reduction of adverse effects from eradication antibiotic therapy by treatments, inhibition and/or control of *Hp* infection, reduction of inflammation; (5) S (Studies): primary studies.

We excluded: (1) studies conducted exclusively on animals; (2) studies conducted on *Hp* cell lines maintained in culture broths; (3) narrative and systematic reviews, case series; (4) studies evaluating the relationship between *Hp* infection and gastric cancer; (5) studies for which the full text was not available; (6) studies concerning data irrelevant to this analysis; (7) duplicate studies.

### 2.3 Study selection

**Figure 1:**
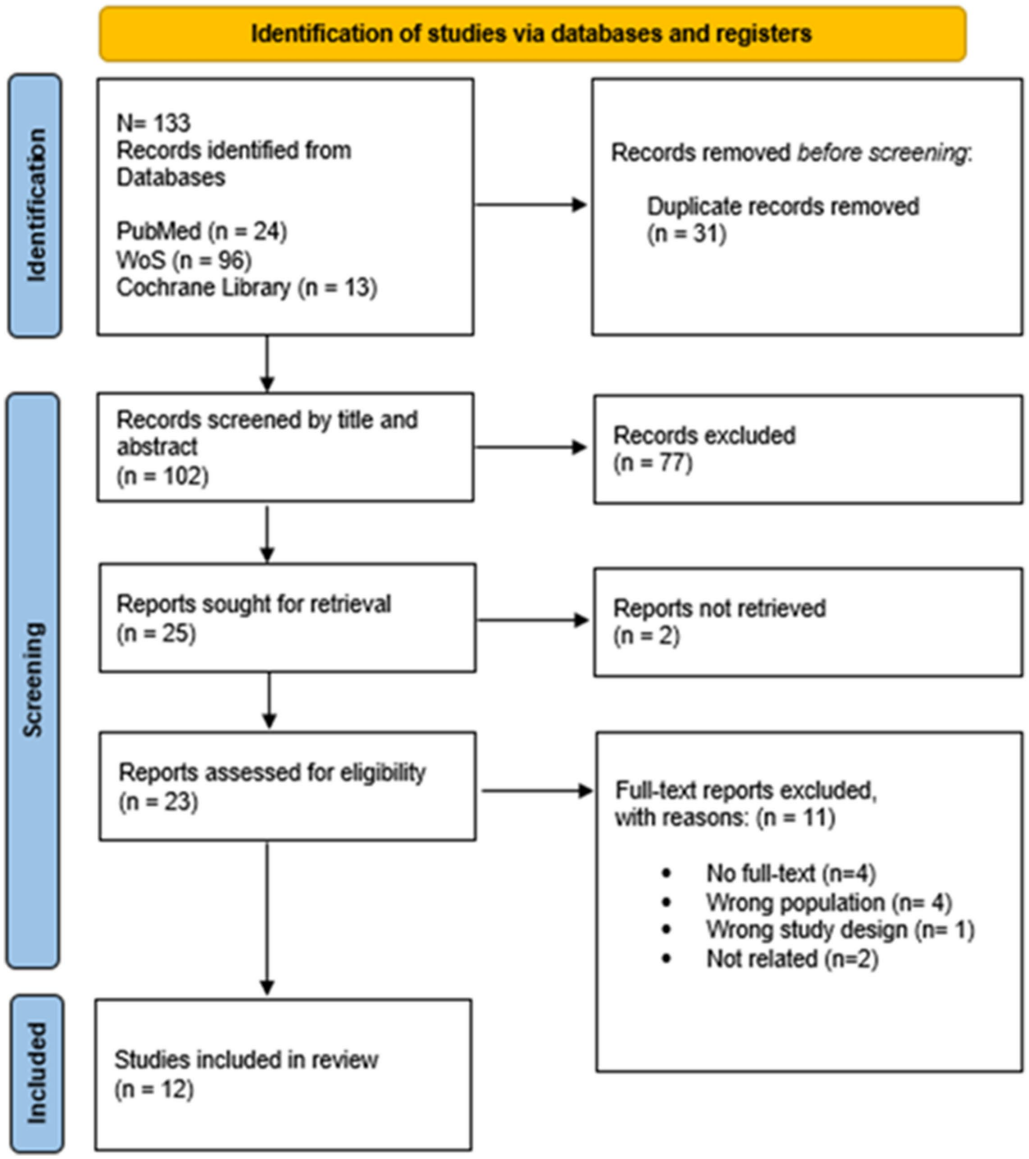
PRISMA flow chart.

The first author (SP) imported the literature into the Rayyan online platform (49). After manually removing duplicates, two reviewers (SP and MR) independently screened the articles, first by title and abstract, then by full-text, to determine eligibility for final inclusion. Discrepancies during screening were resolved by consensus or referred to a third reviewer (AG). In cases where multiple publications were associated with the same studies, a key paper for each study was selected, and then the other associated publications were used for supplementary information during the data extraction process. Sources deemed unsuitable for inclusion at this stage were systematically documented, accompanied by an outline of the reasons for their exclusion (Figure 1). The next phase involved data extraction and cumulative assessment by the reviewers.

### 2.4 Data extraction

Systematic retrieval of relevant information from each article was performed via a standardized data extraction form (Microsoft Excel 2019, Microsoft Corp). The extracted data included the name of the first author, year of publication, geographic region under study, study setting, key characteristics of the study population, study outcomes, and respective quantitative assessment methods. In addition, the data were stratified according to the outcomes investigated the nature of the interventions and the data extraction process was performed by two independent reviewers (SP and MR). Cases of disparity or divergence in data extraction were resolved through extensive deliberation and consensus building.

### 2.5 Data synthesis

In the context of this systematic review, a narrative approach was used to succinctly summarize the data results. The results were described in separate paragraphs based on the type of intervention, to facilitate a comparison between studies. Limited number of studies and data availability across some of the included studies precluded meta-analytic synthesis.

### 2.6 Methodological quality assessment (risk of bias)

For quality assessment, the CASP Randomized Controlled Trial Checklist (50) was employed, which considers 11 aspects grouped into three sections. Specifically, “Section A” provides screening questions about the validity of the basic study design, “Section B” assesses whether the study was methodologically sound, “Section C” provides for the investigation of the results, “Section D” provides for the investigation of local usefulness of results. Responses to the questions were categorized as “Yes,” “No,” or “Cannot tell” with a score of one assigned to affirmative responses.

Two independent reviewers (SP and MR) conducted the assessment process. In cases where disparities arose in assigning scores to individual included studies, these were resolved through thorough reflection and discussion between the two reviewers. Additionally, in cases where consensus could not be reached, the expertise of a third reviewer (AG) was sought to facilitate resolution.

## 3 Results

### 3.1 Study selection

A total of 133 potential records were identified from the searched electronic databases. After removing duplicates (31), 102 articles were reviewed by title and abstract. The full text of 23 studies was assessed for possible inclusion. Eleven studies were excluded for the following reasons: full text unavailable (4), different study population (4), incorrect study design (1), and irrelevant study (2). Twelve studies (51–62), which met all inclusion criteria, were considered in the review (Figure 1).

### 3.2 Study characteristics

Among the included studies, 4 were conducted in Japan (52, 53, 55, 56), 2 in the United States (54, 57), 2 in Korea (51, 58), 2 in Iran (59, 62), 1 in China (61), and 1 in France (60). All included studies are clinical trials, with 2 of them being double-blinded-RCT (58, 61).

The overall quality of the papers was rated as moderate, based on assigned scores of 7/11 (54, 57, 59, 60, 62), 7.5/11 (52, 53, 55, 56), and 8/11 (51, 58, 61) (Table 2).

The study population comprised individuals with confirmed *Hp* infection (51–59, 61, 62), alongside xenografted human cell populations infected with *Hp* (60) (Table 2).

The primary criteria for subject exclusion were consistent across studies, including recent *Hp* eradication therapy (57–59, 61), prior gastric surgery (51, 58, 59), recent use of anti-inflammatory drugs (58), antibiotics (51, 54, 57, 59), PPIs (51, 57, 59), histamine H2-receptor antagonists and bismuth-based formulations (57, 59).

**Table 2:** Studies characteristics.

Author, year	Country	Study design	Population	Intervention	Outcome
A. Yanaka et al, 2009 (56), A. Yanaka et al, 2011 (55), A. Yanaka et al, 2017 (52) A. Yanak et al, 2018 (53)	Japan	RCT	50 *Hp* positive volunteers, whose endoscopy showed no abnormalities other than gastritis.	BS group: 25 *Hp* positive received 70g/day of SGS-rich 3-days-old BS (∼420 μmol of glucoraphanin) for 8 weeks; AS group: 25 *Hp* positive received 70gr of alfalfa sprouts lacking glucoraphanin daily for 8 weeks.	Inhibition a/o control of *Hp* infection; reduction of inflammation.
Y. W. Chang et al, 2015 (58)	Korea	DB-RCT	100 subjects with functional dyspepsia underwent upper gastrointestinal endoscopies, UBT, assessment of MDA and GSH levels. 67 subjects *Hp* positive, 33 subjects *Hp* negative. 11 subjects lost at follow up. 89 Included.	Group A: 33 *Hp* positive take BSES (250 mg standardized broccoli sprout equal to 1,000 mg of sulforaphane). Group B: 28 *Hp* positive take placebo. Group C: 28 *Hp* negative take BSES.	Inhibition a/o control of *Hp* infection; reduction of inflammation.
K. Guo et al, 2022 (61)	China	DB-RCT	533 volunteers with no atrophic gastritis screened by 14C-UBT, of these 138 retested by 13C-UBT, 110 of the 138 retested were included as *Hp* positive.	55 patients randomly allocated to receive 4.5g of AESB (aqueous extract of seed of broccoli) a day, for 2 months before bedtime; 55 patients randomly allocated to receive 4.5g of placebo a day, for 2 months before bedtime.	Inhibition a/o control of *Hp* infection; reduction of inflammation.
P. Mirmiran et al, 2017 (62)	Iran	RCT	362 patients with DM2 assessed for potential inclusion; 191 eligible patients undergo HpSAg test to detect *Hp* infection. Of 108 patients *Hp* positive, 86 were randomly assigned into one of the 3 groups.	Group A (STT *n*=33) received omeprazole 20mg, clarithromycin 500mg, and amoxicillin 1.000mg, twice daily for 14 days; Group B (BSP *n* = 28) received 6 g/day broccoli sprout powder for 28 days; Group C (STT + BSP *n* = 25) received standard triple therapy for 14 days plus 6 g/day broccoli sprout powder for 28 days.	Inhibition a/o control of *Hp* infection; reduction of inflammation.
M. V. Galan et al, 2004 (54)	Michigan	RCT	Patients with evidence of active *Hp* infection established by gastric biopsy or stool antigen testing.	9 patients random assigned to receive 7, 14, or 28 g of broccoli sprouts on an empty stomach twice daily for 7 days.	Inhibition a/o control of *Hp* infection a/o changes in adverse effects from eradication antibiotic therapy by treatments.
A. R. Opekun et al, 2005 (57)	Texas	CT	12 volunteers *Hp* positive at UBT.	5 volunteers *Hp* positive received 135 gr of fresh, finely minced juvenile broccoli tips (135 μmol of glucoraphanin) added to ½ cup commercial plain yogurt; 7 volunteers *Hp* positive received 120ml of Tibetan yogurt whey twice daily for 3.5 days.	Inhibition a/o control of *Hp* infection.
X. Haristoy et al, 2003 (60)	France	RCT	22 xenografts, derived from mature human gastric epithelium and implanted in nude mice, uniformly infected with *Hp*-26695 type.	Intervention group received a solution of 0.5 ml of sterile water with 0.5% acetonitrile containing 7.5 μmol of sulforaphane (1.33 mg) once daily for 5 consecutive days. Control group received an identical solution without sulforaphane once daily for 5 days.	Inhibition a/o control of *Hp* infection.
Z. Bahadoran et al, 2014 (59)	Iran	DB-RCT	362 patients with DM2 assessed for potential inclusion; 191 eligible patients undergo HpSAg test to detect *Hp* infection. Of 108 patients *Hp* positive, 86 were randomly assigned into one of the 3 groups.	Group A (*n* = 33) received standard triple therapy with omeprazole 20mg, clarithromycin 500mg, and amoxicillin 1.000mg, administered twice daily for 14 days; group B (*n* = 28) received 6 g/day broccoli sprout powder for 28 days; group C (*n* = 25) received standard triple therapy for 14 days plus 6 g/day broccoli sprout powder for 28 days.	Interaction of treatments with eradication antibiotic therapy.
Y. W. Chang et al, 2020 (51)	Korea	RCT	Patients with *Hp* positive chronic gastritis or peptic ulcer disease, no clarithromycin resistance.	Group A (61 patients): pantoprazole 40mg, amoxicillin 1g, clarithromycin 500mg twice daily/7 days. Groups B (61 patients) & C (61 patients): same PPI-based triple therapy for 7 days. Additionally, a capsule of probiotics or sulforaphane provided three times daily for 4 weeks to patients in groups B and C, respectively.	Interaction of treatments with eradication antibiotic therapy a/o changes in adverse effects from eradication antibiotic therapy by treatments.

### 3.3 Findings by intervention

#### 3.3.1 Interaction of treatments with eradication antibiotic therapy and/or changes in adverse effects from eradication antibiotic therapy by treatments

In an Iranian RCT (59) in total, 362 patients diagnosed with type 2 diabetes were initially assessed for potential inclusion in the study. Stool samples were collected from the 191 eligible patients to detect *Hp* infection using the HpSAg test. Of the 108 patients identified with *Hp* infection, 86 were randomly assigned to treatment: group A (*n* = 33) received standard triple therapy (STT) with omeprazole 20 mg, clarithromycin 500 mg, and amoxicillin 1.000 mg, administered twice daily for 14 days; group B (*n* = 28) received 6 g/day broccoli sprout powder (BSP) for 28 days; group C (*n* = 25) received standard triple therapy (STT) for 14 days plus 6 g/day broccoli sprout powder (BSP) for 28 days. After accounting for losses at follow-up or treatment discontinuation, 77 of the 86 randomized patients completed the study (STT = 28; BSP = 25; STT + BSP = 24). The eradication rates of *Hp* after the treatment were 85.3, 36.0%, and 83.3 (assessed by UBT) and 89.3, 56.0, and 91.7% (assessed by HpSAg test) respectively in the STT, BSP, and STT + BSP groups. The levels of HpSAg were significantly lower in STT and STT + BSP groups compared to the BSP group, but there were no significant differences in serum PGI, PGII, and PGI/PGII ratio between the three groups. In a Korean trial (51) patients diagnosed with chronic gastritis or individuals with peptic ulcers and *Hp* infection were recruited for the study. Diagnosis of *Hp* infection was confirmed through either rapid urease testing or UBT. Additionally, patients underwent clarithromycin resistance testing and excluded from the study if resistant. The subjects (*n* = 183) were randomly allocated into three treatment groups. Group A (*n* = 61) received triple therapy consisting of 40 mg of pantoprazole, 1 g of amoxicillin, and 500 mg of clarithromycin administered twice daily for 7 days. Group B (*n* = 61) received the same triple therapy regimen for 7 days along with probiotics (Saccharomyces boulardii capsules, 3 × 10^10^ colony-forming units/g). Group C (*n* = 61) received triple therapy for 7 days in addition to sulforaphane supplementation (capsules containing 250 mg of standardized broccoli sprouts, yielding 1.000 μg of sulforaphane) 3 times daily for 4 weeks. The eradication rates -as determined by intention-to-treat (ITT) and per-protocol (PP) analyses- were 85.2 and 89.6% in Group A, 81.9 and 89.2% in Group B, and 86.8 and 96.3% in Group C. No significant difference in eradication rates emerged by treatment group, (ITT analysis: A vs. B, *p* = 0.744; A vs. C, *p* = 1.000; PP analysis: A vs. B, *p* = 0.313; A vs. C, *p* = 0.273). There was also no significant variation in the overall frequency of adverse events (taste disorders, diarrhea, headache, epigastric pain, nausea, and urticaria) among the three groups, although group B showed a lower frequency of gastrointestinal disorders than group A (comparison test A vs. B, *p* = 0.339). The study, therefore, does not demonstrate statistically significant effects resulting from supplementary therapy with probiotics or sulforaphane. In line with those findings, in a RCT conducted in Michigan (54) at baseline, 4 patients reported symptoms (abdominal discomfort such as pain, nausea, and bloating); one patient declared symptom improvement, one no change, and one worsening of symptoms following broccoli sprout therapy.

#### 3.3.2 Inhibition and/or control of *Hp* infection and reduction of inflammation

A Japanese RCT, reported in 4 different publications (52, 53, 55, 56), was conducted to assess the efficacy of a treatment protocol on a cohort of 50 individuals infected by *Hp*, presenting with gastritis as the sole endoscopic abnormality. Participants were divided into two groups: the treatment group (Broccoli Sprouts BS group = 25) which involved the daily consumption of 70 g (∼420 μmol) of glucoraphanin-rich 3-day-old germinated broccoli sprouts for 8 weeks, and the control group (Alfalfa Sprouts AS group = 25) which ingested an equivalent quantity of alfalfa sprouts lacking glucoraphanin, glucosinolates, or isothiocyanates for the same duration. Blood and stool samples of participants were collected at baseline (0 weeks), mid-intervention (4 weeks), post-intervention (8 weeks), and follow-up (16 weeks, corresponding to 8 weeks after the intervention period), and used to evaluate, respectively, PGI, PGII and the PGI/PGII ratio, and the presence of *Hp* stool antigen using HpSAg-ELISA. During the intervention, there were significant reductions of PGII, compared with baseline, only in the BS group, but these returned to baseline values 2 months after the intervention. There was also a significant increase in PGI/PGII ratio in the BS group. HpSAg levels measured in the BS group were significantly lower during the intervention than at baseline but returned to baseline 2 months after the intervention. The placebo group receiving AS showed no significant change in HpSAg. Of the 25 subjects in the BS treatment group, 8 had HpSAg values below the cutoff (0.100) at the end of the 8-week treatment period. In 6 of these subjects, the HpSAg values became positive again at 8 weeks after cessation of BS consumption, and the values for the remaining two subjects became positive again 6 months after intervention, indicating that BS treatment significantly reduced *Hp* colonization during the treatment but did not result in complete eradication (52, 53, 55, 56).

A Chinese RCT (61) enrolled a total of 533 volunteers screened by 14C-UBT for *Hp* infection. Among these, 138 were retested by 13C-UBT for *Hp* infection to exclude false positives. Subsequently, 55 individuals were randomly allocated to the AESB (aqueous extract of seed of broccoli containing 16.31% of glucosinolate, 14.10% glucoraphanin) group, while another 55 were assigned to the placebo group. Participants ingested either 4.5 g of AESB or an equivalent amount of placebo, visually indistinguishable, daily for 2 months before bedtime. Blood samples were obtained following an overnight fasting period to evaluate plasma levels of PG I and PG II and the PGI/PGII ratio, plasma concentrations of inflammatory cytokines, including IFN-*γ*, TNF-*α*, CRP, IL-17A, IL-1β, IL-8, IL-18, and G-17. Normal reference ranges for PG and G-17 were defined as PG I > 70 ng/mL, PGR > 3, and G-17 levels between 1 and 7 pmol/L. The 13C-UBT was performed also at 60 days. No significant differences were observed in any of the inflammatory cytokines between the two groups at baseline and at 60 days follow-up; IL-8 and IL-18 at 60 days were reduced in the AESB groups compared to baseline, but only IL-8 significantly. IFN-γ decreased in both the placebo and AESB group at 60 days, but the reduction was significant only in AESB group. In the placebo group, PGR increased significantly at 60 days compared to baseline, but no significant differences were found in PG I, PG II and G-17 before and after treatment. In contrast, PGR, PG I and G-17 significantly decreased in AESB group at 60 days compared to baseline, but no significant difference was found in PG II after AESB treatment. The eradication rate in the AESB group was greater than in the placebo group at 60 days, but not significantly (11.11% vs. 3.70%, *p* = 0.270) (61). These promising preliminary results suggest that AESB treatment may contribute to prevent *Hp*-induced gastric cancer by reducing inflammation levels (mainly IL-8 and IFN-*γ*).

A RCT (62), additionally conducted in Iran in the framework of the previously mentioned RCT (59) with which shared the *Hp* eradication rate evaluation, examined the possible effect of high SFN broccoli sprout powder versus STT on serum levels of NO metabolites (NOx) as an indicator of systemic NO synthesis, in patients with *Hp* infection. There was a significant decrease in serum NOx levels in BSP (25.2 vs. 51.6 μmol/L) and STT + BSP (31.9 vs. 44.3, μmol/L) groups after intervention. The median, interquartile range, change percent of serum NOx levels were −3.7 (−29.4, 20.3), −42.7 (−57.1, −16.1), and −27.1 (−55.7, −0.92), in the STT, BSP, and STT + BSP groups, respectively. Data suggest that the use of high-SFN broccoli sprouts may attenuate undesirable overproduction of NO in *Hp*-infected patients.

In a Korean DB-RCT (58), 100 participants with functional dyspepsia and non-atrophic erythematous gastritis, without clarithromycin resistance, were randomly assigned in a double-blind manner. Participants underwent upper gastrointestinal endoscopies, UBT, and evaluations of malondialdehyde (MDA) derived from lipid peroxidation, and reduced glutathione (GSH) before and after the intervention. Follow-up assessments were performed within 1 week of taking the broccoli sprout extract supplements (BSES). 67 subjects out of 100 tested subjects were *Hp* positive and were randomly assigned to take BSES capsules (34 subjects) containing 250 mg (1,000 mg of sulforaphane) standardized broccoli sprout extract or placebo (33 subjects) for 4 weeks. 33 *Hp* negative subjects received BSES. Following losses to follow-up, 89 subjects were included, grouped as 33 BSES *Hp* positive (Group A), 28 placebo *Hp* positive (Group B) and 28 BSES *Hp* negative (Group C). In the *Hp* positive BSES group, no statistically significant differences were found in ΔUBT values before and after treatment. Only in one subject did the ΔUBT value reduce to less than 50% of the baseline level. Overall, at baseline, no statistically significant differences were detected in MDA and reduced GSH concentrations in the mucosa between the *Hp* positive and *Hp* negative group, but a significant decrease in MDA concentrations in the mucosa was observed in BSES *Hp* positive subjects compared to before treatment. Furthermore, a significant decrease in MDA concentrations was noted in all subjects treated with BSES, regardless of *Hp* infection status. At baseline, GSH concentrations in the 61 *Hp* positive subjects were lower, but not significantly, than those in the 28 *Hp* negative subjects.

#### 3.3.3 Inhibition and/or control of *Hp* infection

In a trial conducted in Texas (57) the study population was comprised of volunteers with *Hp* infection. After a baseline UBT, volunteers received 135 grams of broccoli tips (equivalent to 135 μmol of glucoraphanin) added to ½ cup commercial plain yogurt, at breakfast, lunch and supper for servings (3.3 days). A final UBT was done 4 h after the last dose. This study also evaluated the effect of freshly made Tibetan yogurt whey in treating human *Hp* infection; volunteers consumed 120 mL of whey twice daily for 3.5 days. There was no significant difference in UBT results before and after a high dose of broccoli (mean UBT results before treatment = 15.8/mil, mean UBT results after treatment = 19.4/mil). Furthermore, there was no significant decrease in the UBT of participants who took Tibetan yogurt whey (UBT before treatment = 35.5/mil ±12.8, UBT after treatment = 40.7/mil±12.2).

In a French study (60), 22 xenografts, derived from mature human gastric epithelium and implanted in nude mice, were uniformly infected with *Hp*-26695 type. 2 weeks post-infection, mucus samples were collected from the xenografts and cultured on blood agar plates to evaluate colonization levels. Additionally, three biopsies were obtained to detect *Hp* and assess gastritis severity. The xenografted animals were subsequently randomized into two groups of 11 each. In the intervention group, a solution comprising 0.5 mL of sterile water with 0.5% acetonitrile containing 7.5 μmol of sulforaphane (approximately 1.33 mg) was administered via catheter once daily for 5 consecutive days. Conversely, the control group received an identical solution devoid of sulforaphane via the same route and schedule for 5 days. 1 month following the cessation of treatment, eradication of *Hp* was noted in 8 out of 11 grafts subjected to sulforaphane treatment. Conversely, the control group exhibited no variation in bacterial concentrations within the mucosa. These findings illustrate the potential for human gastric xenografts to effectively eradicate *Hp* following short-term administration of sulforaphane at a dose of 1.33 mg/day per xenograft.

In another RCT conducted in Michigan (54) eligible patients were identified based on evidence of active *Hp* infection confirmed through gastric biopsy or stool antigen testing. Through random assignment, 9 patients were allocated to receive doses of broccoli sprouts totalling 7, 14, or 28 grams administered on an empty stomach twice daily for 7 days. Stool antigen testing was conducted immediately post-treatment completion and on day 35 following the final sprout dose. Patients who remained *Hp* antigen-negative at day 35 underwent 14C-UBT. The PPI therapy was suspended for 2 weeks before testing. Following each treatment, patients completed a questionnaire regarding symptoms and the palatability of broccoli sprouts. The HpSAg test, conducted immediately following the completion of therapy (day 8), revealed that 7 out of 9 patients (78%) tested *Hp* negative. By day 35, 6 out of 9 patients (67%) remained negative, comprising 2 with negative UBT results, 2 with positive UBT results, and 2 with indeterminate UBT results. Immunohistochemical staining of gastric biopsies confirmed the absence of *Hp* in a patient with an indeterminate test result. Eradication of *Hp* was observed in one patient from each of the three dosage groups (14, 28, and 56 grams/day). Those who did not respond to the study regimen were offered standard antibiotic-based anti-*Hp* therapy. Results seem to suggest that consumption of oral broccoli sprouts was temporally associated with eradication of *Hp* infection in three of nine patients.

## 4 Discussion

The primary objective of this study was to gather empirical evidence regarding the consumption of Brassicaceae and the effects of its constituent sulforaphane, focusing particularly on its potential benefits in addressing human *Hp* infection. Our findings delve into the plausible synergistic interactions between metabolites derived from Brassicaceae, particularly sulforaphane, and antibiotic therapy in addressing *Hp* infection, as well as the role of these metabolites in mitigating *Hp*-induced inflammation and modifying the adverse effects associated with eradication antibiotic therapy.

### 4.1 Summary of findings

Our evidence shows that consumption of Brassicaceae appears to reduce *Hp* colonization (52, 53, 55, 56). Results on *Hp* eradication are controversial and highlight the variability in outcomes. A RCT conducted in Texas (57) suggests that broccoli itself, administered at least as part of a meal, is unlikely to be useful for the treatment of *Hp* infection. This finding aligns with existing literature (63) and is likely due to the lower sulforaphane content in broccoli compared to broccoli sprouts. An RCT conducted in Michigan (54), reported that broccoli sprout treatment led to *Hp* eradication in some patients, but without a clear dose–response relationship. Three RCTs have shown (51, 59) a higher, though not statistically significant, eradication rates, respectively combining Standard Triple Therapy with sulforaphane-compared to Standard Triple Therapy alone- or administering aqueous broccoli seed extract- instead of placebo (61). Beyond eradication rates, the potential protective effects of sulforaphane against *Hp*-induced gastric mucosal damage are noteworthy. A Korean RCT (58), found that consumption of broccoli extract supplements did not affect the density of *Hp* infection, but significantly prevented lipid peroxidation in the gastric mucosa and protected against *Hp*-induced gastritis. Indeed, the evidence (62) from our review revealed significant reductions in NOx levels in all intervention groups. Elevated NOx levels play a critical role in the development of gastric mucosal inflammation, gastritis, and gastric cancer (64, 65). The most pronounced reduction was observed in the BSP group that had received 6 g/day of broccoli sprout powder for 28 days alone or combined with standard triple therapy (62).

The literature robustly demonstrates that the production of ROS and iNOS, along with the resultant increase in NO levels due to *Hp* exposure, can lead to genetic alterations (66, 67). Notably, mutations in p53, induced by elevated NO levels associated with *Hp* infection, occur progressively as the gastric mucosa transitions from gastritis, through intestinal metaplasia and dysplasia, to GC (67, 68). Furthermore supporting this, a Japanese study (52, 53, 55, 56) found that treatment with broccoli sprouts led to a reduction in PGII levels, whose high levels are associated with gastric mucosal inflammation following *Hp* infection (69–71), along with a significant increase in the PGI/PGII ratio. Those findings highlight the potential protective role of sulforaphane against *Hp*-induced oxidative damage to the gastric mucosa, mitigating the inflammation that underlies carcinogenesis. In contrast, an RCT conducted in Iran (59) found no significant changes in serum levels of PGI, PGII, and the PGI/PGII ratio before and after treatment between the groups studied, potentially due to the low amount of broccoli sprout powder administered and the limited duration of treatment. Further affirmation of the potential anti-inflammatory efficacy of sulforaphane is evidenced in the RCT undertaken in China (61), which observed a notable decrease in pro-inflammatory cytokines associated with gastric mucosal injury, peptic ulceration, and gastric malignancy (21, 72, 73) after 2 months of daily AESB dosage. Our systematic review also indicates that the administration of sulforaphane derivatives or probiotics did not significantly alter the incidence of adverse events typically associated with antibiotic therapy, such as taste disorders, diarrhea, headache, epigastric pain, nausea, and urticaria (51). Moreover, the RCT conducted in Michigan (54), highlighted the unpredictable nature of sulforaphane derivatives’ efficacy in ameliorating antibiotic therapy-related side effects, with not concord outcomes in symptom improvement among patients.

As delineated in preceding investigations (41), metabolic derivatives of Brassicaceae exhibit a notable bacteriostatic effect on *Hp* strains conserved in Brucella broth. Our data indicate that the administration of high-dose sulforaphane appears to be efficacious in eradicating *Hp* in short-term human gastric xenografts (60).

Despite the known anticarcinogenic effects of glucosinolates (74), it cannot be excluded that high-dose extracts of Brassicaceae may have undesirable effects, such as hepatocellular damage (75), irritation and increased apoptosis in gastric cells, especially at high concentrations (76). Furthermore, thiocyanates in large quantities formed by hydrolysis of glucosinolates by myrosinase can induce goiter (77). This highlights the continued need for studies to elucidate the various potential effects of these molecules in relation to their concentrations.

### 4.2 Limitations and implications for future research

It is imperative to acknowledge certain constraints inherent in this review. The heterogeneity of outcomes across studies may stem from suboptimal adherence to therapy, variations in the administration of PPIs not uniformly investigated, disparate dosages and timings of sulforaphane and its derivatives. Subsequent investigations should aim to identify an “optimal” dosage capable of mitigating infection, inflammation, and antibiotic-related side effects, since our data shows that too low a dosage could be responsible for a lack of effectiveness of the treatment. Concomitant research endeavors should explore alternative interventions, such as probiotics or other plant derivatives like green tea, garlic or liquorice, in demographically congruent populations. Given escalating antibiotic resistance, comprehensive analyses should encompass resistance profiles to employed antibiotics. Furthermore, evaluating the efficacy of sulforaphane and its derivatives in conjunction with quadruple therapy, including probing resistance to metronidazole, warrants attention.

It is imperative for forthcoming studies to incorporate representative sample cohorts to enhance generalizability. To this end, longitudinal methodologies facilitating the longitudinal observation of phenomena and multicentric approaches, fostering diversity in research contexts and participant demographics, are essential.

Despite strict adherence to PRISMA guidelines, potential selection biases could not be entirely mitigated. Regrettably, a quantitative synthesis was unattainable due to disparate outcome measures and assessment tools across included studies. Standardization of treatment protocols in future investigations would facilitate evaluative comparability and enhance interpretive coherence. Our results suggest that although Brassicaceae consumption may reduce *Hp* colonization, complete eradication of the infection is not consistently achieved in all patients or in all doses administered. However, Brassicaceae derivatives such as sulforaphane appear to have beneficial effects in controlling *Hp* infection. Further studies are needed to fully understand the therapeutic potential of these treatments, with prolonged administration of sulforaphane at different doses, possibly after phase I RCTs to identify the optimal dose, in representative samples using cross-sectional studies and/or multicentre approaches. These efforts would contribute to a deeper understanding of the therapeutic potential of sulforaphane in the management of *Hp* infection and maintenance of gastric health, potentially playing a prominent role in the prevention of gastric malignancies.

## 5 Conclusion

In conclusion, our review show that consumption of Brassicaceae and its component sulforaphane may have an impact on human *Hp* infection through its bacteriostatic role and its inflammation-reducing effect. We have observed promising, but contrasting results regarding the synergistic interactions between Brassicaceae metabolites and antibiotic therapy in eradicating *Hp*, as well as their role in alleviating adverse effects associated with the eradication antibiotic therapy for *Hp*-induced conditions. Instead, several pieces of evidence point to potential protective effects against *Hp*-induced gastric mucosal damage and inflammation, which could be useful in counteracting the progression toward gastric cancer.

Further investigation are recommended to better elucidate a potential protective effect of Brassicaceae or plant extracts on *Hp*-associated gastric cancer risk and their addition to eradication treatment such as Standard Triple Therapy, or Quadruple Therapy, also considering antibiotic resistance patterns, as well as the influence of PPI intake.

## Data Availability

The original contributions presented in the study are included in the article/supplementary material, further inquiries can be directed to the corresponding author.

## Footnotes

^1^ http://www.crd.york.ac.uk/PROSPERO
